# Dynamic Modeling of the Impact of Temperature Changes on CO_2_ Production during Milk Fermentation in Batch Bioreactors

**DOI:** 10.3390/foods10081809

**Published:** 2021-08-05

**Authors:** Jožef Ritonja, Andreja Goršek, Darja Pečar, Tatjana Petek, Boštjan Polajžer

**Affiliations:** 1 Faculty of Electrical Engineering and Computer Science, University of Maribor, Koroška Cesta 46, 2000 Maribor, Slovenia; tatjana.petek@um.si (T.P.); bostjan.polajzer@um.si (B.P.); 2 Faculty of Chemistry and Chemical Engineering, University of Maribor, Smetanova Ulica 17, 2000 Maribor, Slovenia; andreja.gorsek@um.si (A.G.); darja.pecar@um.si (D.P.)

**Keywords:** bioprocess engineering, fermentation process, batch bioreactors, dynamical non-linear mathematical model, model identification, particle swarm optimization, simulation

## Abstract

Knowledge of the mathematical models of the fermentation processes is indispensable for their simulation and optimization and for the design and synthesis of the applicable control systems. The paper focuses on determining a dynamic mathematical model of the milk fermentation process taking place in a batch bioreactor. Models in the literature describe milk fermentation in batch bioreactors as an autonomous system. They do not enable the analysis of the effect of temperature changes on the metabolism during fermentation. In the presented extensive multidisciplinary study, we have developed a new mathematical model that considers the impact of temperature changes on the dynamics of the CO_2_ produced during fermentation in the batch bioreactor. Based on laboratory tests and theoretical analysis, the appropriate structure of the temperature-considered dynamic model was first determined. Next, the model parameters of the fermentation process in the laboratory bioreactor were identified by means of particle swarm optimization. Finally, the experiments with the laboratory batch bioreactor were compared with the simulations to verify the derived mathematical model. The developed model proved to be very suitable for simulations, and, above all, it enables the design and synthesis of a control system for batch bioreactors.

## 1. Introduction

### 1.1. Basic Terms and Topic Relevance

Biotechnology is an engineering and science discipline with enormous importance and potential [1]. The world biotechnology market was estimated at USD 369.62 billion in 2016 [2] and USD 414.50 billion in 2017 [3]. For comparison, it was four times larger than the worldwide production of electric motors (USD 96.97 billion in 2017) [4]. The global biotechnology market is projected to reach USD 727.1 billion by 2025 (the global electric motor market is estimated to reach USD 136.5 billion by 2025). Additionally, in many cases, waste from agriculture or renewable raw material is used as source material in the industrial applications of biotechnology. This is the reason that biotechnology is also essential—to lower dependence on fossil fuels and to reduce greenhouse gas emissions. It is expected that the usage of biotechnology for industrial systems can reduce energy consumption by 20%, water consumption by 75%, and carbon dioxide pollution by 50% [5].

Bioprocess engineering is a biotechnology sub-discipline that is responsible for transferring the discoveries of science results into practical processes, systems, or products that can serve the needs of society [6]. Although the production of biopharmaceuticals is the most visible, bioprocess engineering also has a dominant position in the existing fermentation industries responsible for ethanol production (used for beverages, fuel), lactic acid (for milk products), carbon dioxide, hydrogen gas, butanediol (in pharmaceuticals and cosmetics applications), propanediol (for production of biopolymers), succinic acid (in the chemical, pharmaceutical, food and agricultural industries), and aspartic acid (for production of polymers) [7].

Industrial fermentation is the primary bioengineering process. It represents a planned use of microorganisms (bacteria, yeasts, molds, or algae) or cells (animal or plant cells) to make products advantageous to humans [8].

Industrial fermentation is executed in bioreactors. Bioreactors are classified based on their construction and, consequently, their mode of operation. There are three types of bioreactor: batch bioreactors, fed-batch bioreactors, and continuous bioreactors [9]. The characteristic of batch bioreactors is that, during the implemented biological process, the bioreactor’s content has no contact with external substances or organisms. That means it is closed during the operation, and no inlet or outlet from the bioreactor is possible. Such an operation mode allows non-complicated construction of batch bioreactors, which is seen in low production costs and simple maintenance. This is the reason for the prevalence of batch bioreactors. In fed-batch bioreactors, it is possible to add substances during the execution of the fermentation process. All products wait in the bioreactor till the conclusion of the biological process. Continuous bioreactors (or flow bioreactors) enable the inlet and outlet of substances or organisms into/from the reactor as a flow.

### 1.2. Problem Identification and Aim of the Study

A simple mode of operation and an associated undemanding and inexpensive construction and maintenance represent the great advantage of batch bioreactors. However, their great disadvantage is that we cannot add or remove external substances during the operation, and consequently, we cannot control the dynamics of the metabolism. The microorganism, substrate, and fermentation product concentrations change during the fermentation and, in the current batch bioreactors, depend only on the initial concentrations. Although it is not possible to add or remove individual substances during the operation of batch bioreactors, we can change the temperature during the execution of the fermentation in the modern batch bioreactor. This fact led us to the assumption that we could influence the metabolism during the fermentation process in batch bioreactors by changing the inner temperature. To analyze the influence of the changing temperature and to develop a control system that would ensure that the metabolism will follow the reference, we need an appropriate mathematical model. The development of the dynamic mathematical model of milk fermentation in a batch bioreactor, which would describe the influence of the temperature changes on CO_2_ production, was the main goal of our study.

### 1.3. Related Works and Literature Review

Over recent decades, enormous development has occurred in the fields of Design, Synthesis, and Implementation of Automatic Control for Bioprocess Engineering. A thorough review of the relevant literature in the field of Control of Fermentation Processes in Bioreactors is performed in [10]. Interestingly, however, in most studies in the field of Control of Fermentation Processes, a fundamental kinetic mathematical model [11] is still used, both for theoretical and numerical analysis [12,13,14]. The derivation of a parametric mathematical model of the fermentation process, which also describes the influence of temperature changes on the time course of concentrations of microorganisms, substrate, and fermentation product, is a relatively new field. Although there are many studies showing the influence of temperature on the fermentation processes of different species, these are limited mainly to the observation and numerical evaluation of the influence of different (constant) temperatures on the course of the fermentation process and do not result in deriving a parametric mathematical model [15,16,17].

Mathematical modeling of the fermentation process in bioreactors is highly current, as shown by numerous new publications. Reference [18] shows the implementation of the computational fluid dynamics model of a bioreactor. In [19], the stability analysis of a fundamental kinetic mathematical model of a continuous bioreactor is shown. Reference [20] shows how mathematical modeling can be usefully applied in designing and optimizing bioreactors.

The authors of this paper started working on a parametric dynamical mathematical model that describes the impact of temperature variation on the fermentation dynamics in the year 2018. The influence of temperature change on fermentation dynamics was experimentally observed and described first in [21]. In [22], the authors introduce a new supplementary transfer function that considers the impact of temperature variation on a bioprocess. A conventional control system was developed, and a tuning method was proposed on the basis of this model. In [23], the derived mathematical model was used for the optimization of the control system. In all cases, a mathematical model was derived for probiotic beverages’ fermentation.

The mathematical model presented in [21,22,23] is a “hybrid” mathematical model consisting of an autonomous non-linear model and a non-autonomous linear model connected in parallel. The non-linear model describes the influence of initial conditions on the course of fermentation variables, and the linear model is used to model the impact of temperature changes on their responses. The derived model proved to be a very accurate description of what happened during the fermentation process. This model allows efficient analysis of the fermentation process, but it is demanding to design and synthesize control systems due to its complicated structure. In order to increase the compactness of the model and, thus, increase the suitability of the model for the needs of design and synthesis of control systems, we developed a temperature-considered dynamic mathematical model of the milk fermentation process, which is uniform and does not consist of two parallel sub-models. The derived model is presented in this article.

### 1.4. Paper Contributions

The premise that the control of the metabolism during the fermentation is possible by changing the temperature in the bioreactor leads to the conclusion that, also for batch bioreactors, we can develop a control system, which will control the fermentation process during the operation. The paper’s first contribution is the confirmation of this premise by experiments on a laboratory batch bioreactor. The logical continuation of this finding is the intention to determine the mathematical model that will allow the design and synthesis of the control system. The paper’s second contribution is the derivation of the appropriate mathematical model. The developed model is required to accurately describe the phenomenon during the fermentation process, and, at the same time, the model must be structurally suitable for the development of the control system.

## 2. Materials and Methods

In our study, we focused on the dynamic modeling of the fermentation process in batch bioreactors. Therefore, the subsequent chapters describe in more detail the considered fermentation process and the batch bioreactor, along with the necessary equipment for measurements.

### 2.1. Fermentation Process

The presented study considers milk fermentation with kefir grains. Traditionally, kefir is produced by inoculating kefir grains, which are a mixture of proteins, polysaccharides, mesophilic, homofermentative, and heterofermentative lactic acid streptococci, thermophilic and mesophilic lactobacilli, acetic acid bacteria, and yeast. Fermentation of the milk by the inoculum proceeds for ca. 24 h, during which time homofermentative lactic acid streptococci grow rapidly, initially causing a drop in pH. This low pH favors the growth of lactobacilli but causes the streptococci number to decline. The presence of yeasts in the mixture, together with fermentation temperature (21–23 °C), encourages the growth of aroma-producing heterofermentative streptococci. As fermentation proceeds, the growth of lactic acid bacteria is favored over the growth of yeasts and acetic acid bacteria.

Before the fermentation, kefir grains (40 g) were activated for 5 successive days, washed daily with cold water and put into 500 mL of fresh pasteurized whole fat milk at room temperature. To start the fermentation, 500 mL of fresh pasteurized whole fat milk was preheated in the fermenter to the desired temperature and then inoculated with 40 g of active kefir grains. For the presented fermentation, the full activated (5 days activation) kefir grains were used. Different fermentation processes were obtained by means of differently activated kefir grains.

During the fermentation, carbon dioxide, acetic acid, diacetyl, acetaldehyde, ethyl alcohol, and several other substances are formed, and these give the products their characteristic fresh taste and aroma.

Milk fermentation with kefir grains propagation is an inherently very complex process because of the specific nature of the microbial metabolism, as well as the non-linearity of its kinetics. Therefore, monitoring and control are extremely important to develop models that are able to provide an accurate description of the progress of the process.

### 2.2. Laboratory Equipment

We needed an appropriate laboratory system to determine how the temperature changes affect the time responses of the substance concentrations of the fermentation process. For this purpose, a laboratory system was built, which enabled controlled temperature changes in the bioreactor and dynamic measurement of concentrations of substances during the fermentation process. The laboratory system consists of a batch bioreactor with a heating/cooling thermal system, a measurement system, and a data acquisition system. All parts are described below.

#### 2.2.1. Batch Bioreactor

The fermentation process was primarily analyzed in the computer-controlled laboratory batch bioreactor RC1e from Mettler Toledo (Greifensee, Switzerland). The laboratory bioreactor has additional equipment for the identification of model parameters. The studied batch bioreactor is described in detail in [10,22,23].

#### 2.2.2. Heating/Cooling System

For the identification of the parameters of the mathematical model, an integrated heating/cooling system was used, which controls the temperature of the bioreactor’s mixture. Silicone oil represents the heat transfer agent. The oil is circulated through the bioreactor’s double jacket in a closed circulation system. The temperature control system keeps the temperature of the bioreactor’s contents at the reference value [23]. Compared to the dynamics of the fermentation process, the response of the heating/cooling system is very fast. The identified time constant of the heating/cooling system is approximately 6 min.

#### 2.2.3. Dissolved Carbon Dioxide Measurement System

In the studied fermentation process, the carbon dioxide (CO_2_) dissolved in the bioreactor’s medium is the fermentation product. CO_2_ is a product of the cellular metabolism of microorganisms. Assuming a growth medium with a sufficient carbon source, the measured CO_2_ concentration profile could also be the indicator of the fermentation progress. Thus, dissolved CO_2_ was chosen as the output and identified as an observable variable. The distribution of the CO_2_ in the bioreactor medium is very homogeneous. The sensors for the measurement of CO_2_ concentration are reliable and accurate. The multi-parameter measuring device SevenMulti from Mettler Toledo with modular expansion possibilities was used as a basic unit. For the monitoring of dissolved CO_2_ concentration in liquid media, the SevenMulti apparatus was connected to the ISE51B ion-selective electrode. Electrode potential response to CO_2_ concentration is, in a semilogarithmic scale, a straight line over two decades of the concentration (5·10^−4^ g/L to 2·10^−2^ g/L). A change in temperature causes the electrode response to shift and change slope (temperature variation 5 °C change, slope approx. 1.7%). The detailed principles of the measurement system are described in [10].

#### 2.2.4. Data Acquisition Equipment

For the connection of the SevenMulti basic device and the ion-selective electrode sensor, an expansion module was added to the basic unit. The analog 1st order low pass filter for the elimination of sensor noise is integrated into the expansion module. For the transfer of measured signals from the SevenMulti basic unit to PC, the basic device was equipped with a USB communication module. For the transfer of the measured temperature signal and the reference temperature signal from the heating/cooling system to PC, a dSpace 1103 PPC controller board was utilized. The controller is equipped with 16 bit A/D and D/A converters as well as serial and CAN interfaces [10].

**Figure 1:**
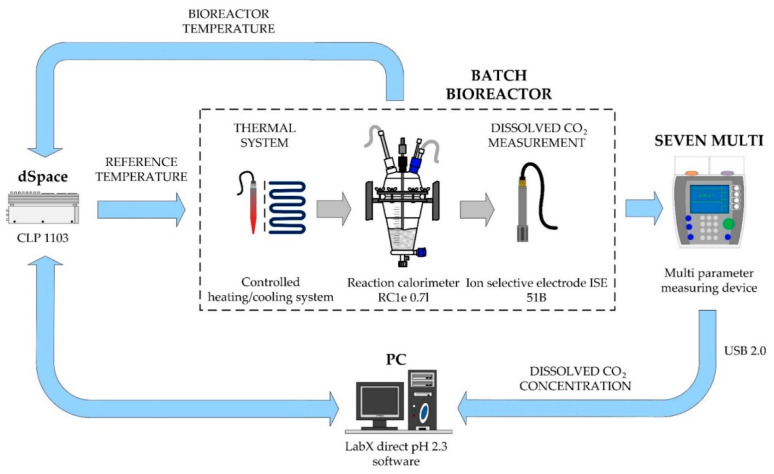
Laboratory system for experiments and identification of the mathematical model.

For the comprehensive measurement of several quantities over a long time period and for the necessary signal processing, software LabX direct pH 2.3, Mettler Toledo (Greifensee, Switzerland) was installed on PC. This is professional equipment used for data logging and data analysis. The selected sampling time was 10 min. This was sufficient due to the slow dynamics of the fermentation process. During the performing the experiments, the sampling time was changed and adjusted to the dynamics of the measured signal. Measured data was saved into Excel, Microsoft Office 365 (USA) documents, transferred into MATLAB R2021a, MathWorks (USA), and processed using MATLAB with its Optimization toolbox functions [10]. A block diagram of the batch bioreactor with measurement system is displayed in Figure 1.

### 2.3. Fundamental Mathematical Model of the Fermentation Process in Batch Bioreactor

Fermentation is a process whereby microorganisms induce a substrate to break down into a fermentation product. Microorganisms, substrates, and fermentation products are present in all fermentation.

The fermentation process is a complex non-linear system with mainly unknown structures and unknown time-varying parameters. In the fermentation process, a multitude of biochemical reactions occurs, representing the kinetics inside a microorganism, in addition to physical transfer rates [9]. The adaptation of the microorganisms to the environment through mutations represents an additional challenge. These are the reasons for the absence of mathematical models, useful from an engineering viewpoint, which would take into account the numerous factors which influence the growth of microorganisms and the execution of the fermentation process.

However, we can find in the literature various mathematical models of different complexity describing the dynamics of fermentation processes in batch bioreactors. These models are mostly based on the mass balances of substances that occur in fermentation. A fundamental kinetic model is written in the state-space form of 3rd order (1)–(3) [10,23],

(1) $${\dot{x}}_{1}\left(t\right)=\frac{\mu_{m}\left(1-\frac{1}{P_{i}}x_{3}\left(t\right)\right)x_{2}\left(t\right)}{S_{m}+x_{2}\left(t\right)+\frac{1}{S_{i}}{\left(x_{2}\left(t\right)\right)}^{2}}x_{1}\left(t\right)$$

(2) $${\dot{x}}_{2}\left(t\right)=-\frac{\mu_{m}\left(1-\frac{1}{P_{i}}x_{3}\left(t\right)\right)x_{2}\left(t\right)}{S_{m}+x_{2}\left(t\right)+\frac{1}{S_{i}}{\left(x_{2}\left(t\right)\right)}^{2}}x_{1}\left(t\right)$$

(3) $${\dot{x}}_{3}\left(t\right)=\left(\alpha \;\frac{\mu_{m}\left(1-\frac{1}{P_{i}}x_{3}\left(t\right)\right)x_{2}\left(t\right)}{S_{m}+x_{2}\left(t\right)+\frac{1}{S_{i}}{\left(x_{2}\left(t\right)\right)}^{2}}+\beta \right)x_{1}\left(t\right)\;$$

where the state space variables and the model parameters are:

*x*_1_(*t*)—the microorganisms’ concentration (g/L),
*x*_2_(*t*)—the substrate’s concentration (g/L),
*x*_3_(*t*)—the fermentation product’s concentration (g/L)
*μ*_m_—the maximum microorganisms’ growth rate (h^−1^),
*P*_i_—the product inhibition constant (g/L),
*S*_m_—the substrate saturation constant (g/L),
*S*_i_—the substrate inhibition constant (g/L),
*A*—the parameter that describes the relation between product yield and microorganism growth, and
*β*—the growth independent constant (h^−1^).

The mathematical model presented in (1)–(3) is autonomous. The model has no input variable. This corresponds to the actual realization of batch bioreactors because they do not have an input quantity to be used for control of the fermentation process. All three substances of the bioprocess are placed in the bioreactor at the start of the fermentation. During the fermentation, it is not possible to add to or remove any of them. The dynamics of the fermentation process depend only on the quantity and quality of substances used and the type of batch bioreactor. Accordingly, the transients of the model are obtained as the response to the initial values of the model variables and depend on the parameters of the mathematical model.

During the fermentation process, the quantity of the microorganisms and product increase, and the amount of substrate decreases. The quantities of substances are measured and applied in the model as concentrations.

In the case of an autonomous fermentation process (the fermentation process that depends only on initial concentrations, where the temperature is constant during the whole fermentation process), all parameters of the fundamental kinetic mathematical model are constant throughout the entire duration of the fermentation process.

The presented fundamental kinetic mathematical model of the fermentation process enables simple and efficient simulation and analysis of the time courses of the concentrations of microorganisms, substrate, and product in the case of different initial concentrations. The main disadvantage of this model is its inability to consider the effect of temperature change on the fermentation process, which is necessary for control system purposes.

This model does not allow the evaluation of the impact of temperature changes on the time courses of concentrations of individual substances during the fermentation process, which is necessary for the design and synthesis of suitable control systems. Therefore, it is necessary to derive a new mathematical model of the fermentation process in the batch bioreactor, which will evaluate the influence of temperature change.

## 3. Results

### 3.1. Experimental Analysis of the Fermentation Process

#### 3.1.1. Responses of the Autonomous Process

**Figure 2:**
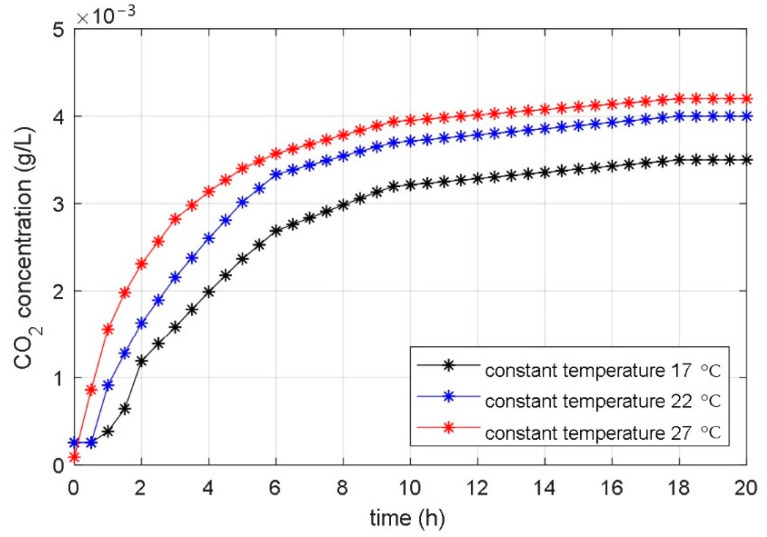
Measured time courses of the CO_2_ concentration during the fermentation processes with different bioreactor temperatures.

First, an analysis was carried out of how the diverse constant temperatures affect the time course of the CO_2_ production. The same initial values for all substances of the fermentation process were used in all experiments. The bioreactor has a constant temperature during the whole fermentation process. The obtained results were expected, meaning that a more responsive fermentation course and a greater end amount of the CO_2_ concentration were achieved at a higher constant temperature. The equal findings were obtained in experiments with different fermentation processes. Figure 2 shows the measured time courses of the CO_2_ for the various constant temperatures of the studied bioreactor.

#### 3.1.2. Responses of the Non-Autonomous Process

**Figure 3:**
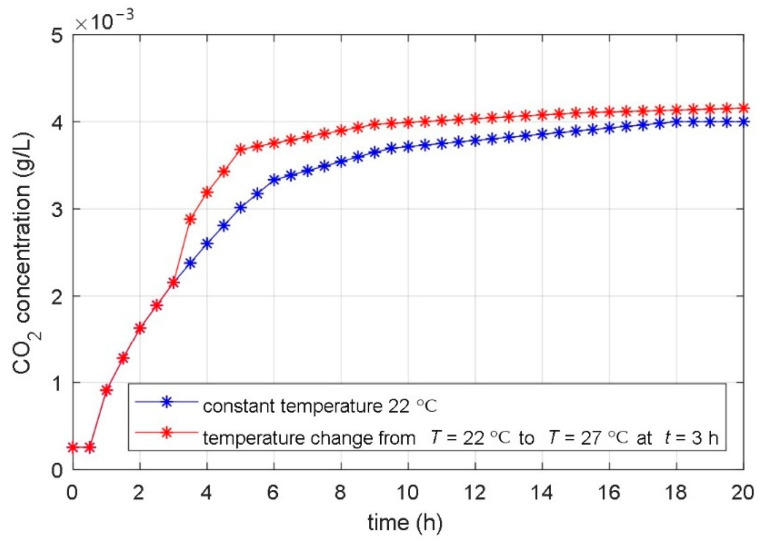
Measured time courses of the CO_2_ concentration during the fermentation processes with constant and changeable bioreactor temperatures.

In the second phase, the impact of the temperature changes during the fermentation was studied. In this phase, many tests were also carried out (different amplitudes, slopes, signs, and different moments of temperature change). It was visible from all experiments that an increase in the temperature speeds up the fermentation, and a temperature decrease decelerates it. The influence of the step change of the temperature on the time course of the CO_2_ concentration is presented in Figure 3. The figure shows the transient response of the CO_2_ concentration when temperature changes from 22 °C to 27 °C. The time course of the CO_2_ for the constant temperature (T = 22 °C) during the entire fermentation was also added to this graph.

### 3.2. Analysis of Correlation between Parameters and Responses of Mathematical Model

**Figure 4:**
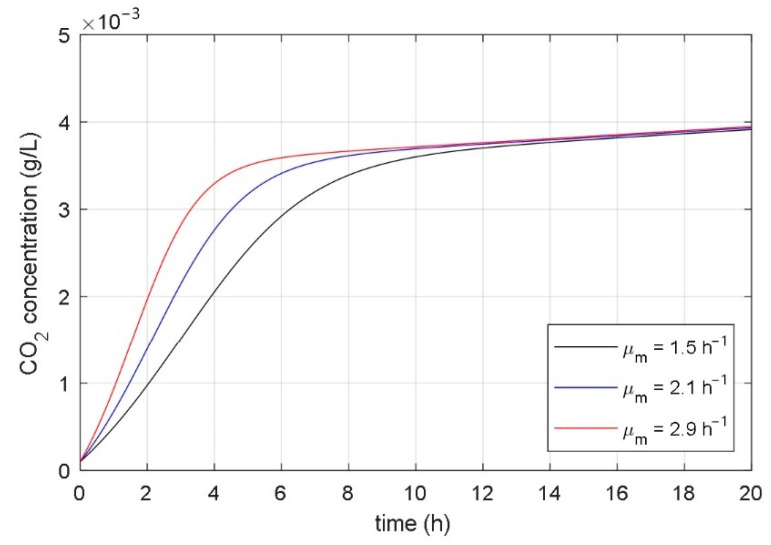
The impact of the maximum microorganisms’ growth rate *μ*_m_ on the time course of the CO_2_ concentration.

**Figure 5:**
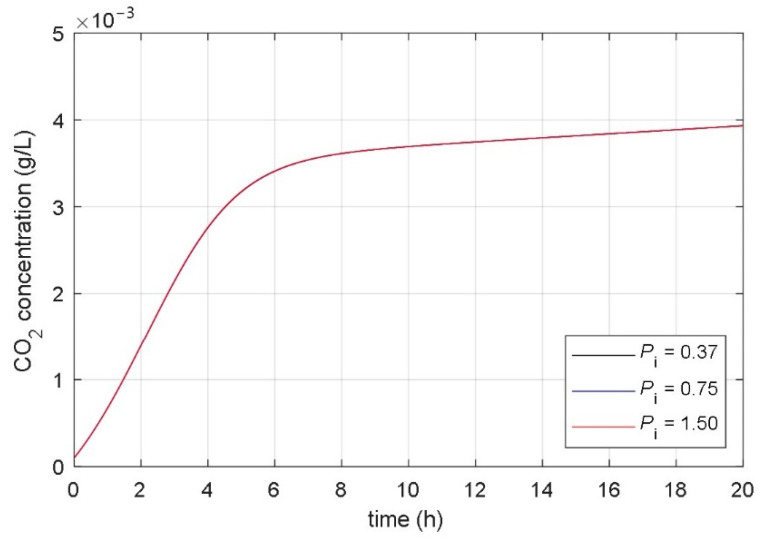
The impact of the product inhibition constant *P*_i_ on the time course of the CO_2_ concentration.

**Figure 6:**
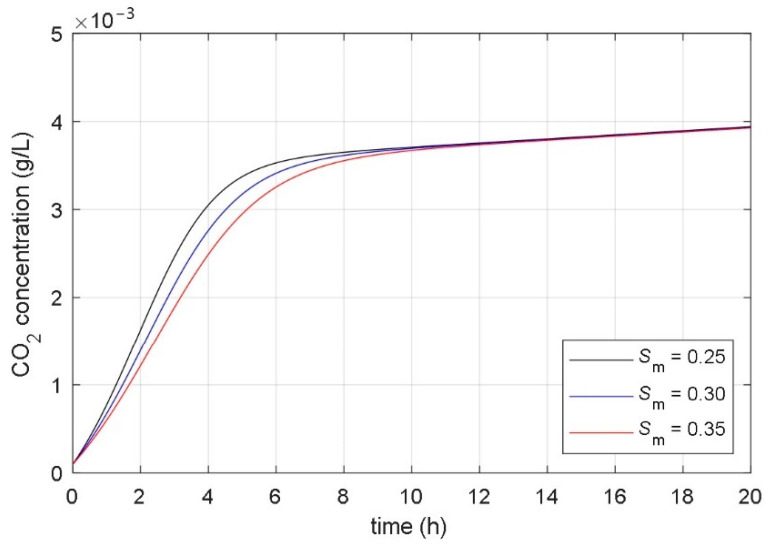
The impact of the substrate saturation constant *S*_m_ on the time course of the CO_2_ concentration.

**Figure 7:**
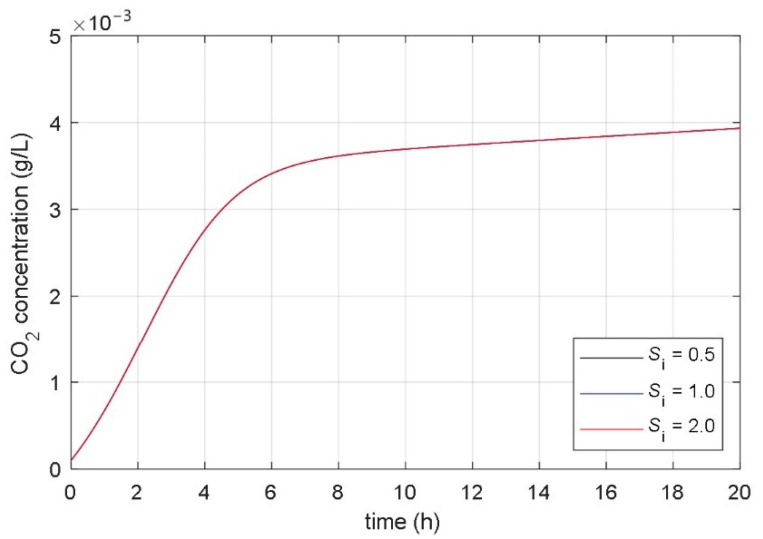
The impact of the substrate inhibition constant *S*_i_ on the time course of the CO_2_ concentration.

**Figure 8:**
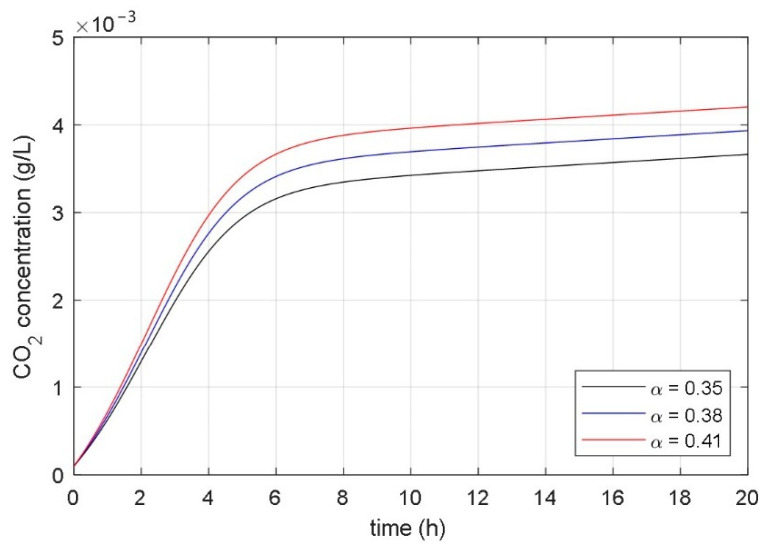
The impact of the parameter *α* on the time course of the CO_2_ concentration.

**Figure 9:**
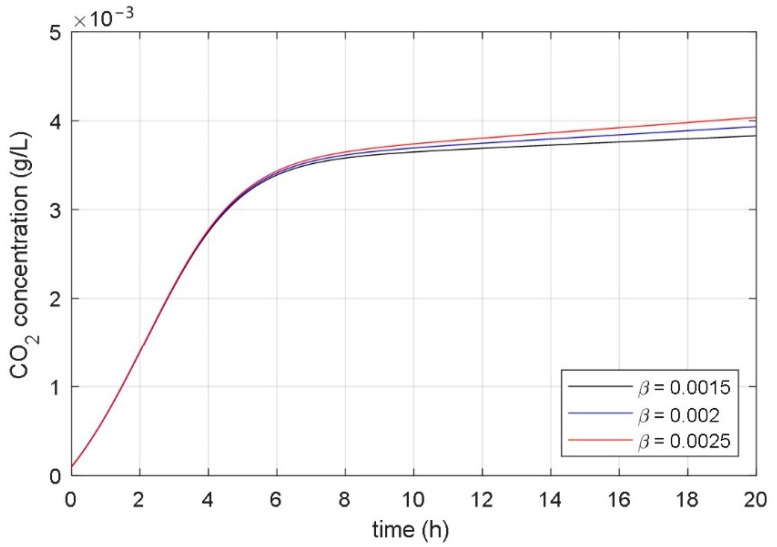
The impact of the parameter *β* on the time course of the CO_2_ concentration.

The aim of the paper was to find a non-autonomous mathematical model whose input variable would be the desired temperature of the heating/cooling system, and the model’s state variables would be the concentrations of microorganisms, substrate, and fermentation product. The starting point for the development of such a model was the idea that the temperature in the bioreactor affects the parameters of the associated mathematical model. As a basis for the development of a new mathematical model, the presented fundamental kinetic mathematical model (1–3) was used. The correlation between the fermentation process and the parameters of the fundamental kinetic mathematical model (1–3) was determined using systematic and extensive simulations. It has been shown that it is significant to analyze the impact of the model’s parameters on the time course of the CO_2_ concentration of the fermentation product (variable *x*_3_(*t*) in (1)–(3)). Figure 4, Figure 5, Figure 6, Figure 7, Figure 8 and Figure 9 show the influence of all mathematical model parameters (1)–(3) on the time course of the CO_2_ concentration, separately. To obtain these results, modified values of the parameters of the mathematical model were presumed. Constant values of the model’s parameters were used during the particular simulation of the fermentation process. Results are shown in the following Figures:

- the impact of the maximum microorganisms’ growth rate *μ*_m_ on the CO_2_ concentration *x*_3_(*t*) is shown in Figure 4,
- the impact of the product inhibition constant *P*_i_ on the CO_2_ concentration *x*_3_(*t*) is shown in Figure 5,
- the impact of the substrate saturation constant *S*_m_ on the CO_2_ concentration *x*_3_(*t*) is shown in Figure 6,
- the impact of the substrate inhibition constant *S*_i_ on the CO_2_ concentration *x*_3_(*t*) is shown in Figure 7,
- the impact of the parameter of the product yield in consequence of microorganism growth *α* on the CO_2_ concentration *x*_3_(*t*) is shown in Figure 8,
- the impact of the independent product yield *β* on the CO_2_ concentration *x*_3_(*t*) is shown in Figure 9.

The goal of the present work was to find the connection between the parameters of the mathematical model and the dynamics of the fermentation process. We were interested in discovering which parameter of the mathematical model had a high impact on the fermentation process dynamics. Based on mathematical models of different fermentation processes in different batch bioreactors, we carried out an extensive systematic numerical analysis. The analyzed fermentation processes have similar impacts of parameter changes on fermentation process dynamics. The main conclusions of all simulations were:

- the impact of the product inhibition constant *P*_i_ and the substrate inhibition constant *S*_i_ on the fermentation process transient and steady-state is very small,
- the substrate saturation constant *S*_m_ has a very small impact on the fermentation process steady-state and a small impact on the fermentation process transient,
- the parameter that describes the product yield that is independent of the microorganisms’ growth *β* has a very small impact on the fermentation process transient and a small impact on the fermentation process steady state,
- the maximum microorganisms’ growth rate *μ*_m_ has a significant impact on the fermentation process transient and very small impact on the fermentation process steady-state,
- the parameter that describes the relation between product yield and microorganisms’ growth *α* has a significant impact on the fermentation process steady-state and very small impact on the fermentation process transient.

Determination of parameters that have an impact on the dynamics and statics of the fermentation process is also possible using sensitivity analysis [24,25,26]. Sensitivity analysis allocates model uncertainty to the various sources of uncertainty, facilitating the targeted reduction of output uncertainty. Sensitivity analysis methods have provided numerous interesting results in a wide variety of different applications. Its advantage is visible especially in systems with a large number of parameters.

### 3.3. Temperature-Considered Model of the Fermentation Process in Batch Bioreactor

From the comparison of the experimental and simulation results, we can see that the temperature changes have a similar effect on the experimentally obtained courses of the fermentation product (Figure 2) as changing the parameters *μ*_m_ and α on the numerically calculated courses (Figure 4 and Figure 8). Based on the results in Section 3.1 and Section 3.2, we can conclude that the temperature change significantly affects parameters *μ*_m_ and α, while it does not have a large effect on other parameters. It can be seen from Figure 4 that the maximum microorganisms’ growth rate *μ*_m_ affects the fermentation speed but does not affect the steady-state value of the fermentation product achieved at the end of fermentation. In contrast, the parameter α affects the steady-state value of the fermentation product and does not significantly affect the transient phenomenon, as shown in Figure 8.

This means that in the case of variable temperature in the bioreactor, the parameters *μ*_m_ and α will no longer be constant, but their values will change according to the temperature in the bioreactor. Therefore, we added a differential equation to the fundamental mathematical model of the fermentation process, with which we calculate the temperature in the batch bioreactor. The temperature in the bioreactor is described by a new variable *x*_4_(*t*). Instead of constant parameters *μ*_m_ and α, we introduce temperature-dependent parameters *μ*_mϑ_(*t*) and α_ϑ_(*t*). In [27], it was shown that there is a static relationship between temperature and the parameters of the mathematical model. Since the temperature during the fermentation varies in a relatively small range (too high or too low temperatures can damage the microorganisms), we used a linear dependence between temperature deviation and model parameters deviation. Consequently, we expressed temperature-dependent parameters *μ*_mϑ_(*t*) and α _ϑ_(*t*) with following static functions:

(4) $$\mu_{m\vartheta }\left(t\right)=\mu_{m}\left(1+k_{\mu m}\left(x_{4}\left(t\right)-\vartheta_{0}\right)\right)$$

(5) $$\alpha_{\vartheta }\left(t\right)=\alpha \;\left(1+k_{\alpha }\left(x_{4}\left(t\right)-\vartheta_{0}\right)\right)$$

where the new parameters in the linear static equations are:

*x*_4_(*t*)—the temperature in the bioreactor (°C),
*ϑ*_0_—the temperature of the bioreactor’s contents at the beginning of the fermentation process (°C), where normally *ϑ*_0_ is equal to the outside temperature,
*k_µm_*—the coefficient that outlined the effect of the temperature changing on the maximum microorganisms’ growth rate *µ*_m_ (°C)^−1^,
*k_α_*—the coefficient that describes the impact of the temperature change on the parameter that describes the relation between product yield and microorganism growth (°C)^−1^,
*μ_mϑ_*(*t*)—the temperature-dependent maximum microorganisms’ growth rate (h^−1^), and
*α_ϑ_(t)*—the temperature-dependent parameter which expresses the connection between product yield and microorganism growth (h^−1^).

The new temperature-considered model was obtained by supplementing the fundamental model with an additional differential equation for calculating the temperature in the bioreactor and by replacing the constant parameters *μ*_m_ and α with temperature-dependent ones, as described by the Equations (4) and (5). The derived mathematical model is expressed with the following equations:

(6) $${\dot{x}}_{1}\left(t\right)=\frac{\mu_{m}\left(1+k_{\mu_{m}}\left(x_{4}\left(t\right)-\vartheta_{0}\right)\right)\left(1-\frac{1}{P_{i}}x_{3}\left(t\right)\right)x_{2}\left(t\right)}{S_{m}+x_{2}\left(t\right)+\frac{1}{S_{i}}{\left(x_{2}\left(t\right)\right)}^{2}}x_{1}\left(t\right)$$

(7) $${\dot{x}}_{2}\left(t\right)=-\frac{\mu_{m}\left(1+k_{\mu_{m}}\left(x_{4}\left(t\right)-\vartheta_{0}\right)\right)\left(1-\frac{1}{P_{i}}x_{3}\left(t\right)\right)x_{2}\left(t\right)}{S_{m}+x_{2}\left(t\right)+\frac{1}{S_{i}}{\left(x_{2}\left(t\right)\right)}^{2}}x_{1}\left(t\right)$$

(8) $${\dot{x}}_{3}\left(t\right)=\left(\alpha \;\;\frac{\left(1+k_{\alpha }\left(x_{4}\left(t\right)-\vartheta_{0}\right)\right)\mu_{m}\left(1+k_{\mu_{m}}\left(x_{4}\left(t\right)-\vartheta_{0}\right)\right)\left(1-\frac{1}{P_{i}}x_{3}\left(t\right)\right)x_{2}\left(t\right)}{S_{m}+x_{2}\left(t\right)+\frac{1}{S_{i}}{\left(x_{2}\left(t\right)\right)}^{2}}+\beta \right)x_{1}\left(t\right)$$

(9) $${\dot{x}}_{4}\left(t\right)=\frac{1}{T_{\vartheta \mathrm{cs}}}\left(u\left(t\right)-x_{4}\left(t\right)\right)$$

where additionally to the symbols in (1)–(5):

*u*(*t*)—indicates the reference temperature of the bioreactor’s temperature control system (°C), and
*T_ϑcs_*—is the time constant of the simple 1st order model of the controlled heating system (h).

The newly developed model considers the influence of temperature on the transient and steady-state of the fermentation process, which is why we named this model the temperature-considered model. The developed enhanced model (6–9) represents the transition between different models (1–3).

When developing and testing the temperature-considered model, instead of linear expressions (4) and (5), we tried different non-linear analytical expressions to describe the dependence of the model parameters on the temperature in the bioreactor. Tested functions were identified in such a way that they enable good fitting of the mathematical model’s responses to the measured trajectories [21]. The selected linear functions represent a good compromise—they are easy to identify and, at the same time, allow a good description of the progress during the fermentation process.

The significant advantage of the temperature-considered model (6–9) is its ability to allow an analysis of the impact of temperature changes on the fermentation process dynamics and steady-state characteristics. The derived mathematical model (6–9) is in structure partially similar to the basic fundamental model (1–3), but in its functionality the newly derived model (6–9) is incomparable to the fundamental model (1–3). The fundamental model (1–3) is an autonomous model and allows only simulations of responses of the concentrations to the initial values of fermentation substances. On the contrary, the model (6–9) represents a non-autonomous model of non-linear differential equations, which additionally enables the calculation of the courses of concentrations of bioreactor substances in the case of temperature variations in a bioreactor. Therefore, model (6–9) is suitable for the design of fermentation control systems based on temperature changes in the bioreactor, which is not possible with model (1–3).

### 3.4. Parameters Identification of the Temperature-Considered Model

The parameters of the mathematical model *μ*_m_, *P*_i_, *S*_m_, *S*_i_, *α*, *β, k_µ__m_*, *k_α_*, and *T_ϑ__cs_* (all in Equations (1)–(9)) depend on the quality and quantity of the substances, and of the external operating parameters. In the case of unchanged external conditions, the parameters remain more or less constant during the fermentation process [21]. The parameters of the mathematical models for the fermentation processes in the laboratory or industrial bioreactors can be calculated by different optimization methods from the measured trajectories of the bioreactors’ substances. In our study, the particle swarm algorithm was used to obtain the parameters of the mathematical model.

Particle swarm is a population-based algorithm [28]. Particle swarm optimization (PSO) is originally attributed to [29]. During the optimization the swarm of particles varies throughout the selected area. The optimization algorithm calculates the objective function at each step. After their calculation, the algorithm sets the new particles’ velocities. The algorithm moves each particle to the best-founded location. PSO is a metaheuristic procedure that may provide a sufficiently good solution to an optimization problem, in case of few, incomplete, imperfect, or no assumptions about the problem being optimized. PSO can search very large spaces of candidate solutions. PSO does not use the gradient of the problem being optimized, which means that it does not require that the optimization problem be differentiable. However, PSO does not guarantee that an optimal solution is ever found.

Functions from MathWorks MATLAB/Optimization Toolbox library were used for faster realization of the PSO for the calculation of the model’s parameters. Matlab function particleswarm.m is based on the algorithm described in [29], using modifications suggested in [30,31]. Details of the PSO algorithm in the particleswarm.m function are written in [32].

The optimization was accomplished from the measured time course of the CO_2_ concentration. For the objective function requires, the error between the measured and calculated response was computed. The integral absolute error (IAE) objective function was implemented to calculate the fitting of the mathematical model with the laboratory batch bioreactor [10].

Because we approximately estimated the range of values of the model’s parameters, we constrained the area where the algorithm was searching for optimal solutions. The optimization algorithm changed the parameters of the mathematical model (6–9) for so long in order to reach the minimum of the IAE function. Optimization was finished when the relative change of the objective function reached the stopping criterion suggested in the default defined value.

For the determination of the temperature-considered model, it was necessary to identify nine parameters of the mathematical model. PSO can be used to identify all parameters simultaneously, but we can also identify the model’s parameters in several stages. With the help of a systematic trial procedure, we found that the best results are obtained by separating the parameters identification into two phases. In the first phase, the parameters *μ*_m_, *P*_i_, *S*_m_, *S*_i_, *α*, and *β* were identified. In the second phase, the remaining parameters *k_µ__m_*, *k_α_*, and *T_ϑ__cs_* were calculated.

#### 3.4.1. Identification of the Parameters *μ*_m_, *P*_i_, *S*_m_, *S*_i_, *α*, and *β*

In the first stage of the optimization procedure, the parameters *μ*_m_, *P*_i_, *S*_m_, *S*_i_, *α*, and *β* were identified. These represent the fundamental part of the mathematical model, which coincides with the constant temperature environmental conditions. This models the fermentation process’s transient and steady-state behavior resulting from the initial concentrations of the substances in the bioreactor.

First, the initial values of the microorganisms, substrate, and fermentation product for the fermentation process in the studied batch bioreactor were measured. The measured values are written in Table 1.

**Figure 10:**
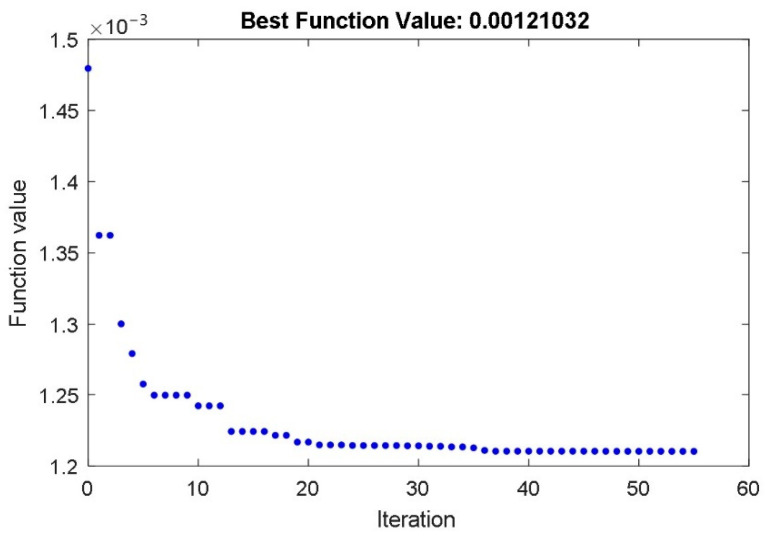
The time course of the IAE objective function during the PSO with the parameters *μ*_m_, *P*_i_, *S*_m_, *S*_i_, *α* and *β*.

After that, the fermentation process was carried out. Only the dissolved CO_2_ concentration needed to be measured. The dissolved CO_2_ concentration represents the fermentation product and is the most important substance in the bioreactor. During the fermentation process, all external conditions (temperature, stirrer’s speed) were constant. The measured time course of the dissolved CO_2_ concentration for constant temperature 22 °C is shown in Figure 2. The PSO with IAE objective function was applied to calculate the parameters of the fundamental part of the model, valid for the constant bioreactor’s temperature. The IAE function was calculated as the integral of the absolute error between the measured course of the dissolved CO_2_ concentration (Figure 2) and the calculated state-space variable *x*_3_(t) of the non-linear mathematical model (1–3). The PSO algorithm changed the values of the identified model *μ*_m_, *P*_i_, *S*_m_, *S*_i_, *α*, and *β* for so long that the objective function reached a minimum. The time course of the objective function during the PSO procedure is shown in Figure 10.

As can be seen from Figure 10, less than 60 iterations were required to calculate the parameters *μ*_m_, *P*_i_, *S*_m_, *S*_i_, *α*, and *β* of the fundamental part of the mathematical model. All identified parameters are presented in Table 2.

#### 3.4.2. Identification of the Parameters *k*_µm_, *k*_α_, and *T*_ϑcs_

In the second stage of the optimization, the parameters *k_µ__m_*, *k_α,_* and *T_ϑ__cs_* were identified. These parameters are important for the supplementary part of the mathematical model, which describes the influence of the variable temperature on the fermentation process transient and steady-state behavior. The parameters *k_µ__m_* and *k_α_* characterize the impact of the variable temperature on the modifying of the parameters *μ_mϑ_*(*t*) and *α_ϑ_(t)*. The parameter *T_ϑ_*_cs_ specifies the temperature time constant that describes the alternating of the temperature in the bioreactor as a result of the reference temperature of the bioreactor’s temperature control system.

**Figure 11:**
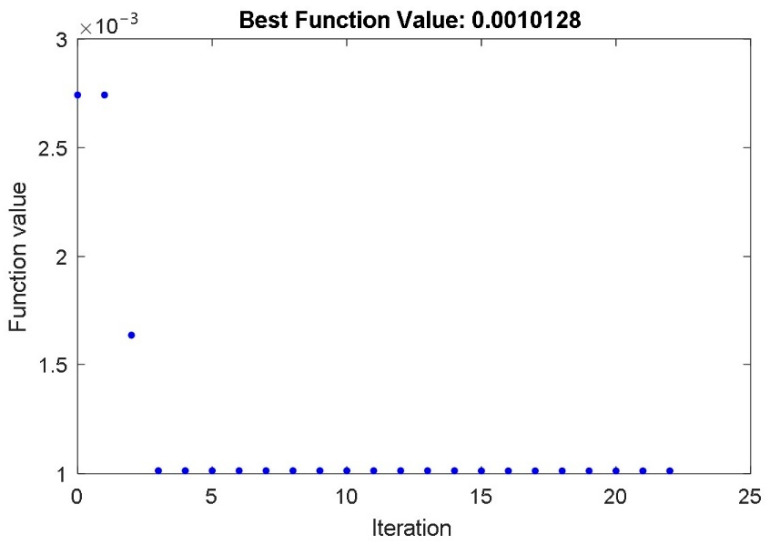
The time course of the IAE objective function during the PSO with the parameters *k_µ__m_*, *k_α_*, and *T_ϑ__cs_*.

To identify these parameters, a fermentation process was carried out again. This time, we changed the reference temperature during the fermentation process. The step change of the temperature from 22 °C to 27 °C arises at *t* = 3 h. The time course of the dissolved CO_2_ during the fermentation with changeable temperature is shown in Figure 3. Again, the PSO with IAE objective function was used for the identification of the model parameters. The IAE objective function was calculated as the integral of the absolute error between the measured course of the dissolved CO_2_ concentration (Figure 3) and the calculated state-space variable *x*_3_(t) of the non-linear mathematical model (3–6). The PSO algorithm changed the values of the identified model *k_µm_*, *k_α,_* and *T_ϑcs_* for so long that the objective function reached a minimum. During the optimization, the parameters *μ*_m_, *P*_i_, *S*_m_, *S*_i_, *α,* and *β* have values that were identified in the first stage of the identification procedure (Table 2). The time course of the objective function during the PSO procedure is shown in Figure 11.

Figure 11 shows that less than 25 iterations were required for the calculation of the parameters *k_µ__m_*, *k_α_*, and *T_ϑ__cs_* of the supplemental part of the mathematical model. The identified parameters are presented in Table 3.

To estimate the temperature-considered mathematical model parameters, we can also use the computationally less demanding sensitivity method [24,25,26]. Its advantage is especially evident in models with a large number of parameters.

### 3.5. Simulation Results of the Temperature-Considered Model

**Figure 12:**
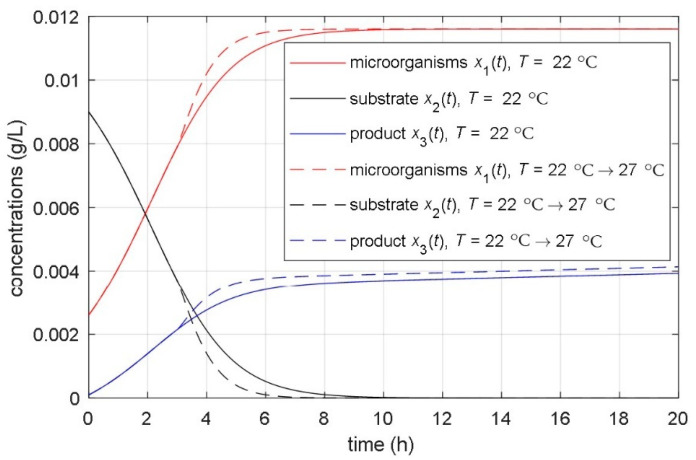
Time courses of the concentrations of the microorganisms, substrate, and product during the fermentation processes with constant and changeable bioreactor temperatures.

The simulation results of the identified temperature-considered model of the fermentation in the laboratory bioreactor are shown in Figure 12. Presented are the time responses of the microorganisms, substrate, and product in the case of constant temperature (solid lines) *T* = 22 °C, and the same variables in the case of the bioreactor’s reference temperature step change from 22 °C to 27 °C in *t* = 3 h (drawn with dashed lines). It is evident that temperature changes generate substantial variations in the dynamics of all quantities of the fermentation process. The time course of the actual temperature in the bioreactor follows the reference temperature step change. The delay in the actual temperature is small—it corresponds to the short time constant of the controlled heating system *T_ϑ_*_cs_ = 0.1 h.

### 3.6. Comparison of Simulations and Experimental Results

**Figure 13:**
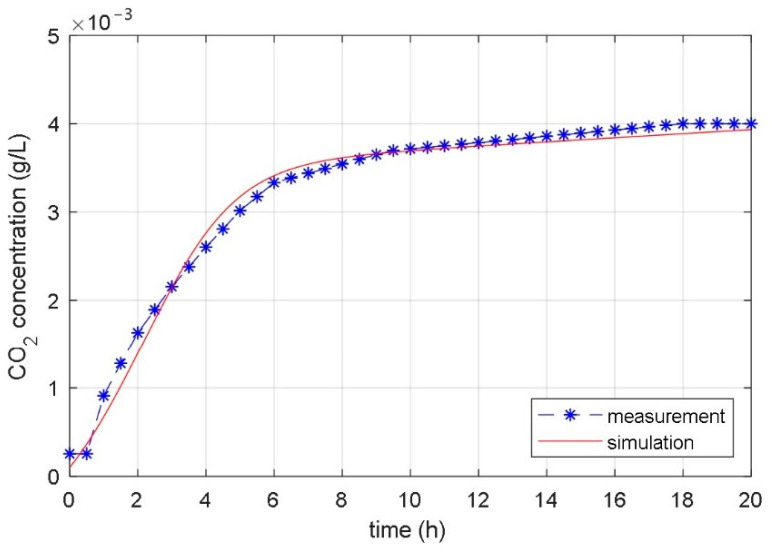
Measured and simulated time courses of the CO_2_ concentration during the fermentation process with constant bioreactor temperature 22 °C.

**Figure 14:**
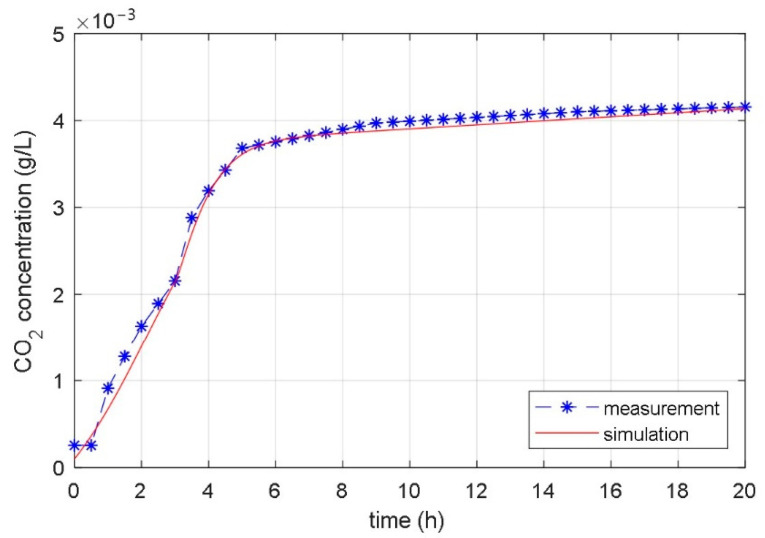
Measured and simulated time courses of the CO_2_ concentration during the fermentation process with changeable bioreactor temperature (step change of temperature from 22 °C to 27 °C occurred at time *t* = 3 h).

**Figure 15:**
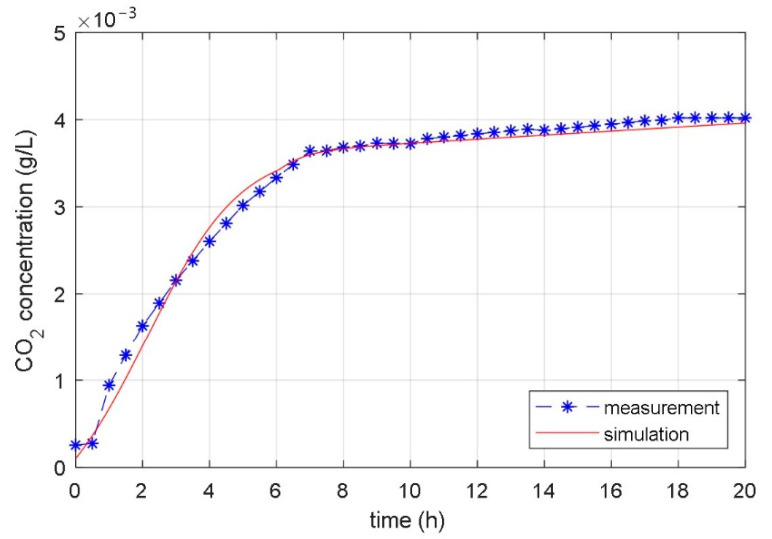
Measured and simulated time courses of the CO_2_ concentration during the fermentation process with changeable bioreactor temperature (step change of temperature from 22 °C to 27 °C occurred at time *t* = 6 h).

The matching of the response of the measured CO_2_ concentration in the laboratory bioreactor with the response of the CO_2_ concentration calculated with the identified temperature considered model is displayed in Figure 13, Figure 14 and Figure 15.

The results of the fermentation process with a constant temperature 22 °C are presented in Figure 13. It can be seen that in the case of a constant temperature, the time course of the produced CO_2_ is continuous. The response of the identified mathematical model matches very well with the measured response of the laboratory bioreactor. Matching is seen in all three phases of the fermentation process: in the starting induction phase, in the successive exponential growth phase and in the subsequent stationary phase and the end dying phase.

Figure 14 shows the results of the fermentation with the changeable bioreactor’s temperature. Note, in the experiment and in the simulations, the step increase of the reference temperature from 22 °C for 5 °C occurred at *t* = 3 h. The consequence of the step change in temperature in the bioreactor is visible in the different slope of the time course at the time of the change. In this case, the matching of the responses of the identified model and the laboratory measurements is also very good.

Figure 14 shows a comparison of simulations and measurements for the input signal, which was previously used to identify model parameters. It makes sense to test the accuracy of the identified mathematical model for a new signal dataset. This is the case where the test input signal is different from the input signal used for identification. Figure 15 shows the results of the fermentation where the step increase of the reference temperature from 22 °C for 5 °C occurred at *t* = 6 h. Simulation and experimental results are presented. As in the previous cases, both responses match very well. The non-continuity in time course, smaller as in the previous case, is seen at *t* = 6 h. Figure 14 and Figure 15 confirm the empirical findings that the effect of temperature change on CO_2_ production is bigger at the beginning of the fermentation (high growing rate) and smaller in the second part of the fermentation process (low growing rate).

The simulation results show that the derived model can justifiably be used for bioprocess analysis, simulations, and control system development. The identification (with optimization techniques) of the parameters of the presented model is not complicated but can take a lot of time. For identification purposes, a fermentation experiment with a constant temperature must first be performed. After that, the fermentation process must be carried out again. This time the temperature must change during fermentation.

## 4. Discussion and Conclusions

The study’s main contribution is the development of a new dynamic model that considers the impact of temperature changes on the fermentation process dynamics and steady-state characteristics. The most important features of the presented temperature-considered model are:

- The derived model is a fourth order non-linear state-space model,
- The model’s input is the reference variable of the heating/cooling system, the model’s state-space variables are the concentrations of microorganisms, substrate and product, and the temperature in the bioreactor,
- The derived model is compact and suitable for the analysis of the fermentation process, for simulations, and for the implementation of the control system.
- The derived model represents a further development of the models presented in the authors’ previous publications [10,21,22]. The advantage of the new model is in its simpler structure—previous models were based on a combination of a non-linear model for calculating the response to initial conditions and a linear model for calculating the response to temperature change.
- The derived model has nine parameters that depend on the biochemical substances and the determination of parameters for a specific bioreactor is possible by identification. For parameters’ identification, it is necessary to carry out the fermentation process at least twice: once with a constant temperature and once with a change in temperature during the fermentation. The paper does not discuss the theoretical possibility of determining the model parameters from the biochemical data. Particle swarm optimization was used for the identification of the mathematical model.
- An important finding obtained from the derived model analysis is that the application of adaptive control theory for non-linear systems is reasonable for developing a control system that will control the fermentation process along the prescribed reference trajectory [33].
- Identification and analysis of the mathematical model were made on different fermentation processes on two different batch bioreactors. The obtained results of a multitude of experiments and calculations confirmed the presented findings.
- The article shows the use of scientific methods for the needs of engineering research. Our expectations are that academics and engineers will use the developed mathematical model for the design and synthesis of advanced control systems.

## Figures and Tables

**Table 1:** Initial values of the fermentation process in the studied bioreactor.

Variable	Value
the initial value of the microorganisms’ concentration	*x*_1_(0) *=* 2.6 mg/L
the initial value of the substrate’s concentration	*x*_2_(0) *=* 9.0 mg/L
the initial value of the product’s concentration	*x*_3_(0) *=* 0.1 mg/L
the initial temperature of the bioreactor’s contents	*x*_4_(0) *=* 22 °C

**Table 2:** Parameters of the Mathematical Model for the Fermentation Process in the Studied Bioreactor.

Parameter	Value
the maximum microorganisms’ growth rate	*μ*_m_ = 2.1 h^−1^
the product inhibition constant	*P*_i_ = 0.75 g/L
the substrate saturation constant	*S*_m_ = 0.03 g/L
the substrate inhibition constant	*S*_i_ = 1.0 g/L
the parameter of the product yield related to microorganisms’ growth	*α* = 0.38 $\frac{g/L}{g/L}$
the parameter of the product yield independent of the microorganisms’ growth	*β =* 0.002 h^−1^
the temperature of the bioreactor’s contents during the fermentation process	*ϑ*_0_ = 22 °C

**Table 3:** Additional parameters of the augmented mathematical model for the fermentation process in the studied bioreactor.

Parameter	Value
the coefficient of the impact of the temperature changing on the maximum growth rate *µ*_m_	*k_μ_*_m_ = 0.14 (°C)^−1^
the coefficient of the impact of the temperature changing on the parameter α	*k*_α_ = 0.03 (°C)^−1^
the time constant of the 1st order model of the controlled heating system	*T_ϑ_*_cs_ = 0.1 h
the temperature of the bioreactor’s contents at the beginning of the fermentation process	*ϑ*_0_ = 22 °C

## Data Availability

The data presented in this study are available on request from the corresponding author.
